# Patients’ perceptions of frequent hospital admissions: a qualitative interview study with older people above 65 years of age

**DOI:** 10.1186/s12877-020-01748-9

**Published:** 2020-09-07

**Authors:** Miaolin Huang, Carolien van der Borght, Merel Leithaus, Johan Flamaing, Geert Goderis

**Affiliations:** 1 Leuven Institute for Healthcare Policy, Department of Public Health and Primary Care, KU Leuven, Leuven, Belgium; 2 Academic Centre for Nursing and Midwifery, Department of Public Health and Primary Care, KU Leuven, Leuven, Belgium; 3 Department of Geriatric Medicine, University Hospitals Leuven, Leuven, Belgium; 4 Department of Chronic Diseases, Metabolism and Ageing, Gerontology and Geriatrics, KU Leuven, Leuven, Belgium; 5 Academic Center for General Practice, Department of Public Health and Primary Care, KU Leuven, Kapucijnevoer 33 Blok J Bus, 7001 3000 Leuven, Belgium

**Keywords:** Frequent flying, Patient perception, Hospital, Qualitative research, Primary care, Older people, Frequent admission

## Abstract

**Background:**

Although ‘frequent flyer’ hospital admissions represent barely 3 to 8% of the total patient population in a hospital, they are responsible for a disproportionately high percentage (12 to 28%) of all admissions. Moreover, hospital admissions are an important contributor to health care costs and overpopulation in various hospitals. The aim of this research is to obtain a deeper insight into the phenomenon of frequent flyer hospital admissions. Our objectives were to understand the patients’ perspectives on the cause of their frequent hospital admissions and to identify the perceived consequences of the frequent flyer status.

**Methods:**

This qualitative study took place at the University Hospital of Leuven. The COREQ guidelines were followed to provide rigor to the study. Patients were included when they had at least four overnight admissions in the past 12 months, an age above 65 years and hospital admission at the time of the study. Data were collected via semi-structured interviews and encoded in NVivo.

**Results:**

Thirteen interviews were collected. A total of 17 perceived causes for frequent hospital admission were identified, which could be divided into the following six themes: patient, drugs, primary care, secondary care, home and family. Most of the causes were preventable or modifiable. The perceived consequences of being a frequent flyer were divided into the following six themes: body, daily life functioning, social participation, mental status and spiritual dimension. Negative experiences were linked to frequent flying and could be situated mainly in the categories of social participation, mental status and spiritual dimensions.

**Conclusions:**

Frequent hospital admissions may be conceived as an indicator, i.e., a ‘red flag’, of patients’ situations characterized by physical, mental, spiritual and social deprivation in their home situation.

## Relevance to clinical practice

Our results suggest that older patients with frequent hospital admissions should be ‘flagged’ because they are in need of special attention from a transmural, multidisciplinary team of social and health care workers. This team should provide a holistic and patient-centered approach, including case management and a program of advanced care planning. This approach may also reduce the number of hospital admissions, which is a hypothesis that is still in need of further research.

## Impact statement

What does this paper contribute to the wider global clinical community?

- Frequent hospital admissions result from complex interactions between patients’ physical and mental condition, attitude, values, social situation and issues with care provision for both primary and secondary care
- Frequent hospital admissions may therefore be considered an indicator, i.e., a ‘red flag’, of patients’ physical, mental, spiritual and social deprivation in their daily living conditions.
- Older patients with frequent hospital admissions should be ‘flagged’ because they are in need of special attention from a transmural, multidisciplinary team of social and health care workers. This team should provide a holistic and patient-centered approach, including case management.

## Background

Hospitalization remains a main source of health expenditure. In 2016, hospital expenditures accounted for 38% of all EU health expenditures [1]. Moreover, from 2006 to 2016, the total number of admissions increased by 28.1% [2]. In addition, unplanned hospital readmissions after discharge occur regularly and induce high costs. In Belgian hospitals, unplanned readmission rates after hospital discharge reach approximately 5.2% [3]. Of particular interest are those patients who frequently use emergency department (ED) services and have frequent hospitalizations. These ‘frequent flyers’ can be defined as patients who have been admitted at least four times in 12 months [4–6]. Although frequent flyers represent barely 3 to 8% of the total patient population in a hospital, they are responsible for a disproportionately high amount of all admissions (approximately 12 to 28%) [7, 8]. The hospitalization of frequent flyers is often more complicated, and after hospital discharge, these patients are twice as likely to be readmitted, often due to comorbidity [3]. Unplanned hospital readmission and unplanned frequent use is considered low-value care [9]. Because of the high associated costs, intervention programs that aim to reduce frequent hospital admissions are becoming a priority [10, 11]. Indeed, several actions seem to reduce unplanned hospital (re)admissions, such as care coordination [12], case management [13], transitional care interventions [14], hospital discharge management plans [15] and even physical activity [16]. However, the group of frequent flyers is heterogenic and can be divided into patients over 65 years of age, patients with psychiatric disorders, patients with chronic conditions and patients with a low socioeconomic status [17]. In this study, we focus on the group older than 65 years of age who often have multiple chronic conditions and unique requirements that are not always met by conventional approaches in health care delivery [8]. Indeed, older people with multi-morbidity are often very vulnerable individuals [18] with a high need for care and increasingly unmet needs [19, 20]. Therefore, it is important to understand the patients’ view of his or her situation, and until now, only a few studies have incorporated the patients’ perspectives [21, 22]. Several studies have demonstrated that some knowledge, for instance, about adverse events and medical errors, is only detected from the patients’ perspective and not by other methods [23]. To fill this gap, this study aims to comprehend the phenomenon of the frequent flyer from a patient’s perspective. The objectives were to obtain an in-depth understanding of the perceived causes of frequent hospital admissions, to identify the perceived consequences of being a frequent flyer and to expose the contextual elements of frequent hospital admissions (perceived causes, contributing factors and perceived consequences) and the interactions between these elements.

## Methods

### Study design

This qualitative study took place at the University Hospital of Leuven (UZ Leuven), which is a tertiary teaching hospital in Belgium. The study had a phenomenological design and was carried out between January 2019 and May 2019. We received ethical approval from The Ethics Committee Research UZ/KU Leuven. The Consolidated Criteria for Reporting Qualitative Research (COREQ) guidelines were followed to provide rigor to the study (see supplementary file 1). For more information about the research team and reflexivity, see supplementary file 2 [24].

### Study population and recruitment

The study population was recruited from the three acute geriatric wards (with a total of 78 beds) of the UZ Leuven Gasthuisberg campus. These acute geriatric wards operate with an interdisciplinary team based on comprehensive geriatric assessment and have a mean length of stay of 11 days. Discharge planning with referral to primary care services is provided for each patient. In regard to the current study, once a week, a nurse working in the geriatric wards identified eligible participants by reviewing the electronic medical records. The inclusion criteria included ‘very frequent’ [25], i.e., at least four, admissions in the past 12 months at the UZ hospital, an age above 65 years and admission at the time of the study. The exclusion criteria were poor hearing function, a known diagnosis of dementia, having delirium, non-Dutch speaking, being in a palliative and/or terminal care setting and having no speech. Eligible participants were approached by the researchers (CVDB and CMH) during their hospital stay and were given both oral and written information. The participants each signed an informed consent form.

### Data collection

An interview guide based on the literature and the objectives of the study was used during the semi-structured interviews (see supplementary File 3). The guide was first pilot-tested on a potential participant whose transcript was included in the final analysis. Depending on the information obtained, the guide was revised to fill in the blind spots of the study. The interviews were audio recorded for subsequent transcription and coding. After each interview, the findings were discussed among the researchers. The overall process was repeated until reaching data saturation, i.e., when no new themes emerged. Finally, no compensation was given to the participants, and no field notes were made.

### Analysis

All the interviews were transcribed verbatim into a text document, anonymized and analyzed following the QUAGOL (Qualitative Analysis Guide of Leuven) guidelines, which serve as an iterative guidance tool for qualitative data analysis [26]. A systematic comparative analysis was used to examine the data. To become as familiar as possible with the interview data, CVDB and CMH each read, analyzed and discussed the content of the transcriptions. Both independently compiled a narrative summary report for each interview that was then summarized in an interview scheme in which the concrete findings were described on a more abstract conceptual level. The interviews with the narrative summary report and the conceptual interview scheme were then reviewed by the other researcher and modified to discuss potential discrepancies. Based on the narrative summary report and the conceptual interview scheme, the codes were discussed and determined.

Subsequently, the coding process started. This process consisted of two parts: preliminary coding and effective coding. In the preparation phase, a list of topics was drawn based on the conceptual interview schedule. The two researchers discussed and compared the codes on similarities and discrepancies until a primary coding structure was established. In this way, a codebook with code definitions was developed. Each researcher independently read the thirteen interviews and compared them with the codebook. The interviews were coded, and comments were made on the decisions made during the coding process to ensure coherence. This ensured that the initial codebook was reviewed and adjusted to the new findings. When the coding of the interviews was completed, the codebook was further refined and divided into two major categories. The first category included codes concerning triggers that cause hospitalizations among the participants. The second category consisted of codes that referred to elements that physically, socially, psychologically and emotionally affect the participants’ state of health. These two categories with codes provided answers to the research questions. Finally, quotes from the interviews were selected by the two interviewers to underline the results. The data were encoded in NVivo version 11 Pro (QSR International’s®).

Finally, a fishbone diagram and spider plot were used to describe all concepts and integrate them into a general conceptual framework that illustrates the findings and provides answers to the research questions. Codes were divided into perceived causes and contributing factors. Perceived causes were viewed as responsible in a direct causal way for frequent flying, whereas contributing factors were viewed as influencing frequent hospital admissions by increasing their likelihood, accelerating the number of admissions in a period of time or affecting the severity of the consequences. Perceived causes were subdivided into underlying causes and triggers. For example, take a situation in which an older patient falls at home; in this example, falling is a trigger since it is the direct cause for admission, while the lack of fall prevention is an underlying cause because it could have prevented the fall. Further, contributing factors were subdivided into medical and nonmedical factors and family factors. The model of Machteld Huber about positive health was used to subdivide the consequences that patients experienced due to their frequent hospital admissions [27, 28]. After the study, the recordings were deleted.

## Results

### Participant characteristics

A total of thirteen interviews were collected. All the interviewees consented to participate in the study. There were no dropouts, refusals or repeated interviews. In some cases, first-degree family members were present. These family members also consented to provide informed written consent. Their answers were also included but were mainly complementary to those of the participants. This inclusion gave further insight into interpreting the participants’ answers, as these relatives often provided additional, relevant information. The interviews usually lasted 40–50 min. No transcripts were returned to the participants. The median age of the participants was 85 years, with a range from 73 to 97 years. The participants were admitted on average four times in the last 12 months. More than half of the participants were women (7/13).

### Perceived causes of frequent hospital admissions

**Fig. 1:**
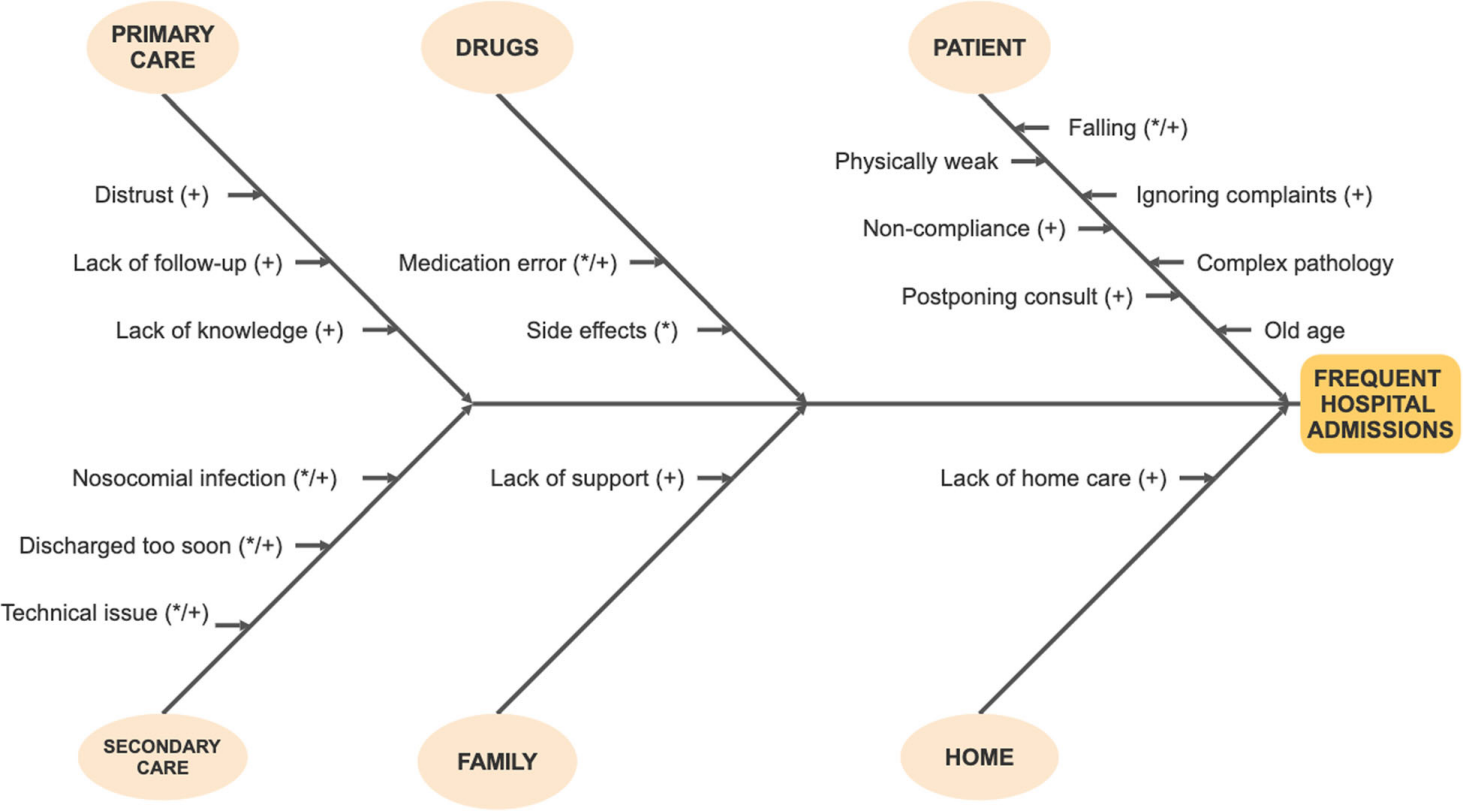
Perceived causes of frequent hospital admissions presented in a ‘Fishbone Diagram’. Legend: * = trigger; + = preventable/modifiable

A total of seventeen perceived causes were identified, which could be divided into the following six different categories: patient, drugs, primary care, secondary care, home and family. Most of the causes occurred in the categories of patient, primary care and secondary care. Each cause was either a trigger or an underlying cause. In total, fewer triggers (6/17) than underlying causes (11/17) were identified. These triggers were found in the categories of drugs, secondary care and patient. In addition, most of the causes could be prevented or modified (13/17). Some participants indicated that if these causes were solved, then admissions would happen less often. Finally, it is noteworthy that all the causes related to medical care are preventable or modifiable. In primary care, admissions were related to underlying causes, and in secondary care, admissions were related to triggers. See Fig. 1.

In the patient category, old age, being physically weak and having a complex pathology were the most common causes, but they were not modifiable. However, falling was found to be a major trigger, and it can be prevented.

*“I have had a hernia with serious episodes and discomfort for 25 years. After cycling with my brother-in-law, I started feeling pain in my toes. They applied traction to my lower back, but that did not help. After a while, they took scans and gave me an injection in my spinal cord. Eventually, a young surgeon performed surgery on me. It was hard because that bulge had progressed for 25 years, and he had to take it out. It did not completely cure me. Afterwards, I was still doing small chores in the garden and in the house, but then I hurt myself. The pain lasted for a long time, and it turned out to be inflammation. So, I had to take anti-inflammatory drugs. Occasionally I took one, and then I had a stomach bleed and Barrett syndrome. Now they want to put 6 stents in my heart. I already have 2, but I do not want any more. In addition, I also have angina pectoris.”*

Participant 11; complex pathology

In addition, causes related to the patient’s behavior were also identified, namely, ignoring complaints, noncompliance and postponing consultation. This behavior was shown to lead to frequent hospital admissions, but it is modifiable or preventable. In some cases, this kind of behavior was induced by the bad experiences of other patients.

*“If I had taken my antibiotics earlier, I might not have caught it chronically. It is because of me and not the clinic.”*

Participant 2; noncompliance

Furthermore, side effects and medication errors also triggered frequent hospital admissions. In theory, they are preventable, but in practice, it is difficult to avoid side effects. The lack of home care and family support were identified, as well as underlying causes of frequent hospital admissions. The need for family support was strongly highlighted during a number of interviews.

*“He [son] does not come to the clinic very often and certainly not because of his clinic phobia; so, he does not come to the clinic, and he does not come to the nursing home much either. (...) Only my daughter is coming to get me now. She knows that she is the only one, and it has started to bother her.”*

Participant 1; lack of family support

Finally, there were medical causes such as lack of follow-up, lack of knowledge, technical issues, too-early discharge and nosocomial infection that could be prevented. In addition, distrust in primary care physicians went hand-in-hand with confidence in specialists. As a result, these participants wanted to be treated by a specialist and no longer by their primary care physicians.

### Contributing factors

Alongside the perceived causes of frequent hospital admissions, contributing factors were also found. A complete overview of all contributing factors can be found in Supplementary File 4. A total of 14 categories were identified and divided into three areas: medical, nonmedical and patient. Appendix 2 gives an overview of all the contributing factors. As expected, some overlap between causes and contributing factors was found according to the expressed nuance of the participants, indicating a continuum in the degree of perceived causal determination.

### Medical

First, the problems with general practitioners pertained to the lack of follow-up, the lack of information, the lack of initiative, the lack of knowledge, the lack of patient involvement, wrong estimation and professional jargon. These factors may lead to a loss of confidence in general practitioners.

Participant 1: *“Well, I have to be honest. It was me who worked towards it [the admission] because the doctors at home did not want to find a solution on their own. They have been saying for a long time that “Yes, they said that in the clinic” but it did not come from them. (…) They have not taken good care of it, like looking for initiatives, treating me or improving my health.”*

Interviewer: *“So you’re saying that you worked towards being admitted?”*

Participant 1: *“Yes, because they could not find a solution there in the retirement home, and here in the hospital they can.”*

Participant 1;

distrust in primary care physicians

going hand-in-hand with confidence in specialists and a lack of initiative

In addition, the availability of general practitioners was also a drawback. The search for a good general practitioner is a difficult task, and even if they have a good one, the participants felt that little time is provided for them.

*“In September, I had a stroke;” at the clinic they said to me that “Your doctor must come to you this evening [Friday].” I came home and I made the phone call. The doctor said, “I do not have time, I will send my colleague... ON MONDAY.”*

Participant 9; general practitioner too busy

Second, in the nursing homes, poor coordination, miscommunication and insufficient care were identified as contributing factors. It was mentioned that physicians do not take enough initiative to address the problems and give insufficient attention to patient involvement. Additionally, incorrect estimations and medication errors have been made in the past. All of this led to distrust towards physicians in nursing homes.

Third, the hospital and specialists were mentioned. On the one hand, fragmented and insufficient care were revealed. For example, it was mentioned that there are too many treating physicians, that responsibilities are passed to others, that double therapy occurs and that overreporting takes place. Patients reported not being treated with a holistic approach and that patient-specific care was lacking. A criticism of the profession was that it is overspecialized. On the other hand, the hospital was also praised by some participants. These patients felt well cared for and had confidence in the hospital. One participant argued for admission because he wanted to be treated by the specialist and not by a primary care physician. In addition, many participants pointed out that although they prefer to be at home, their hospital admissions have been pleasant and professional experiences.

*“It’s laughable. For ENT (ear, nose, throat), it will not take long before there comes one doctor for the nose, one for the throat and one for your ears. One for your left ear and one for your right ear. So far, this is how the specialization is progressing.”*

Participant 11; overspecialization

Finally, polypharmacy was cited in almost every interview. The participants reported taking many medications without having proper knowledge of their indications. Many participants did not prepare their medication themselves but let others prepare it for them, e.g., family members or home nurses. Transmural care also remained an issue, where sometimes no proper care or information were provided after dismissal. As a result, the participants did not know how to continue their treatment after they were discharged.

*“I do not know what I'm getting anymore. At least 10, 11 or 12 pills early in the morning, and in the afternoon about 3, and in the evening about 2-3 pills. Then, also an inhaler.”*

Participant 1; polypharmacy

### Nonmedical

Nonmedical problems included family, home care and social environment. This study identified a lack of home care, neighbor contact and other social contacts. Some participants have no friends left because their friends have already died. Because of this lack, these participants mainly rely on their existing family members. In some families, a lack of support and contact was seen. Some participants have even had a quarrel with their family, and they do not speak to each other anymore. Others disagreed with their relatives about the care they should receive. Finally, the loss of a loved one and widowhood were also mentioned as contributing factors of frequent hospital admissions.

*“I’m just struggling on my own because I cannot ask much of my son. For example, I would never dare to ask him to wash me. (…) The family connection is not that strong.”*

Participant 3; lack of family support

### Patient

Patient-related problems included behavior, physical factors, finances and lifestyle. Regarding the patients’ behavior, some participants mentioned not caring about the medical advice they received and being easily satisfied. Others were interested in their medical condition, but they could not understand everything told to them and were afraid to ask for clarification. There were also participants who would not independently seek help without the permission of their general practitioner.

*“If I do not understand the explanation, I will be silent for sure. If you only know something, that is enough. It is as simple as that.”*

Participant 7; easily satisfied

In addition, there were participants who had postponed their treatment because of the bad experiences of their family members. Some had lied to their physicians about quitting smoking. There were also participants who did not accept home care because they were afraid of strangers. Others were too proud to admit that they cannot do everything on their own and that they need help.

Furthermore, health care also involves high costs, which played a role in the decision making of the patients and their family members. In some situations, the participant did not receive the necessary home care because of the financial barrier of the family. On the other hand, participants with private health insurance were reassured that all the expenses would be covered.

Alcohol and smoking were also important contributing factors. Many participants who had been smoking and consuming alcohol for many years had to quit. That was not an easy task. Some have succeeded in quitting, others have not. Those who did not succeed justified their alcohol consumption/smoking behavior.

*“I also had a few pints at night. That was my sedative. And once you are retired, you have to go to bed early. It was only getting later and later. So, I had another cigarette and another pint. Just beer, no heavy beer. And 25 cl, not 33 cl. Sometimes 5-6 beers a day. I did that regularly. Not to say every day.”*

Participant 11; alcohol and smoking

Finally, it was pointed out that the participants had too much time to think and contemplate. After many years, physicians still did not know the origin of certain complaints. Thus, the participants themselves did not know much about their illness or its background. There were also misconceptions among the participants, for example, that a vaccination makes you immune to the flu virus.

*“There are moments that go well, but there are also days that you think ‘Goddamn, now I get up to go back to sleep afterwards.’ I walk around here [hospital] and at home in circles. (…) There is no future anymore. (…) You have time to think. You have nothing to do but to think. Too much time.”*

Participant 5; too much time to think and contemplate

### Perceived consequences of frequent flyer status

**Fig. 2:**
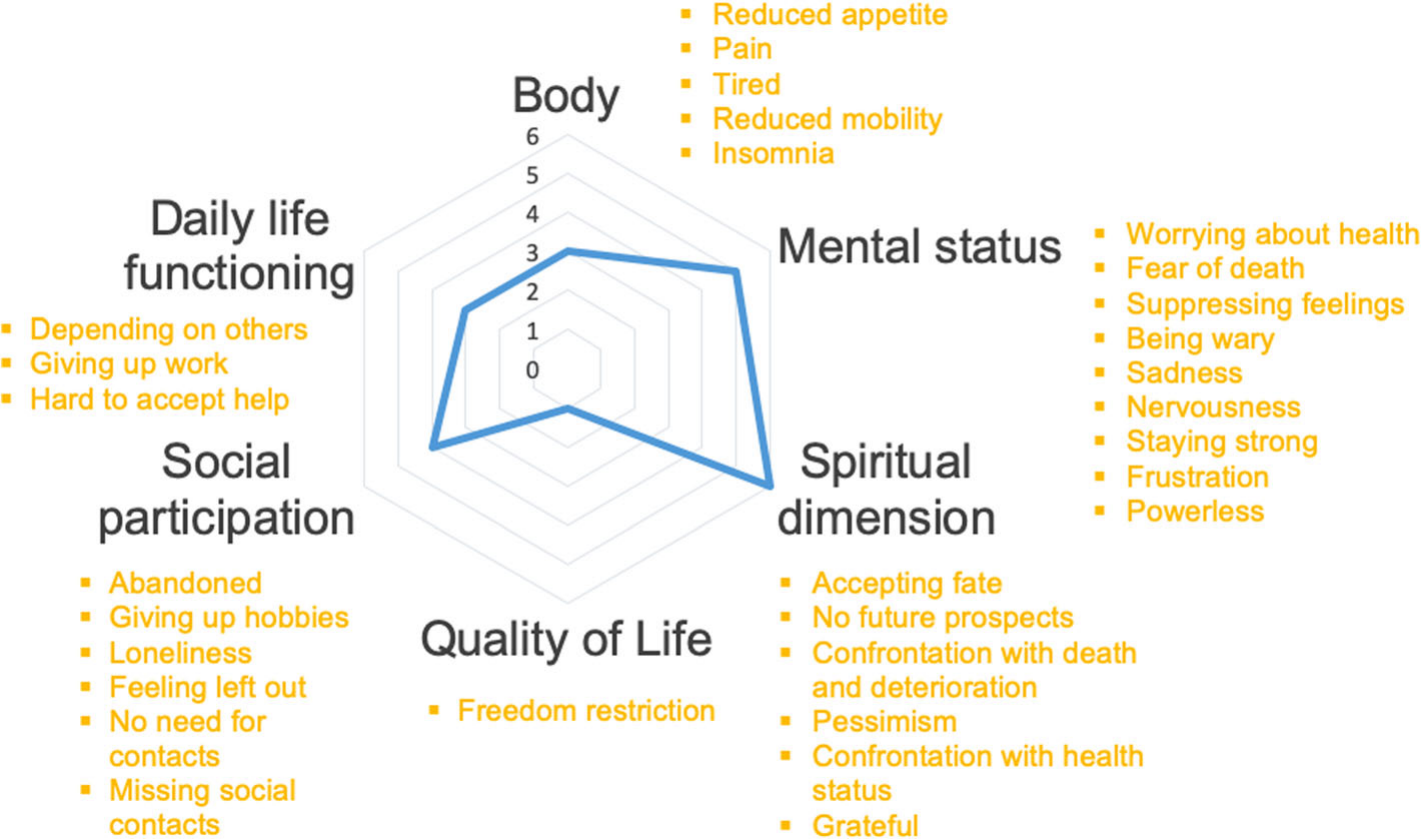
Perceived consequences of frequent flyer status presented in a Radar Chart. Legend: The blue line indicates to what extent the six categories were present in this study

As shown in Fig. 2, the perceived consequences were classified into six categories, namely, body (physical health), daily life functioning, social participation, quality of life, mental status and spiritual dimension. This classification has been made based on the concept of positive health [27, 28]. Regarding the patients’ perceived consequences, all the categories could contain at least 1 item. However, most of the participants identified consequences related to the categories of mental status and spiritual/existential dimension. Moreover, social participation was also an important element.

### Mental status

The participants predominantly described three feelings in the mental status category, namely, powerless, fear of death and frustration. They felt powerless because they experienced a deterioration in themselves. They could not perform activities of daily living by themselves and had become more dependent on others. This led to a difficult feeling of losing control.

The second feeling that often came up was the fear of death. Many participants reported having much time to reflect on everything around them. Thus, they were concerned about their state of health and did not see any improvement in their medical condition. For this reason, the thought of death came to mind more often in these patients.

*“If you have nothing else to do than to wait and sleep, then you hope to wake up the next day because you never know for sure what could happen.”*

Participant 5, fear of death

Many participants were also frustrated about the fact that things were not as easy as they used to be; i.e., they reported a confrontation with physical decline. Especially after their frequent hospital admissions, many participants noticed that they had become considerably weaker over the past year. The most frustrating part was the awareness of their own decay.

Interviewer: *“Was it difficult to resume your daily routine after hospitalization, to get back home?”*

Participant 4: *“Yes, you have to be able to resettle. You want to do a lot of things, but you cannot. That is difficult, very difficult.”*

Participant 1: *“No, but I have experienced that I have confronted myself with the fact that I could perform less than before.”*

Participant 4 and 1; frustration

### Spiritual/existential dimension

The spiritual/existential dimension illuminated pessimism, gratefulness, confrontation with death and accepting fate as important perspectives. Some participants were pessimistic because they had lost sight of future prospects and the meaning of life. They did not see their state of well-being as improved and only felt deteriorated. Some of them were still struggling with those unpleasant thoughts, while others had evolved to accept their fate.

*“Well, today the food was good and that is all matters. If you have nothing else to do than to wait, you have time to think. You have nothing else to do than to think and yes...the next day I hope to wake-up again. There is not much future for me.”*

Participant 5; spiritual dimension

In addition, many participants were grateful for the help and the support they received from their families. Other participants received very little support or had even lost contact with their family. This lack of contact affects their state of mind, and some even indicated that it could play a role in their frequent hospital admissions.

Furthermore, several participants were confronted with death. Many of their friends and acquaintances had already died.

### Social participation

In regard to social participation, missing social contacts and giving up hobbies were mainly discussed. A number of participants indicated that they missed certain social contacts in their lives. Some participants felt lonely and were more dependent on others for transport, which made it more difficult to maintain their social life. Additionally, many of their friends had died, which meant that their social circle was getting smaller and that they had fewer remaining relationships as a result.

*“I cannot drive a car anymore. I am going to see my friends a lot less. It is harder for me to go there. I live on Sint-Maartensstraat, and if I want to go to the station with my friends, because they like to have a beer there, that will not work. I would also like to go, but that is no longer possible.”*

Participant 11; missing social contacts

On the other hand, there were participants who did not need much time for social connection. They were in search of rest time and thus preferred to be at home with their families.

*“I do not really have the need for extended social contacts anymore. I like to stay together at home with my husband. I am very happy with that.”*

Participant 4; less preference for social contacts

Additionally, some participants felt abandoned. They used to visit many friends who were sick at the time, but now that the roles are reversed, they do not see many visitors. Other participants felt abandoned by their family, for example, because of a family quarrel or for no particular reason.

*“I'm very disappointed. In the past, when I could still get around and heard that someone was ill, I always went to visit them. Always. And they do not come to me now.”*

Participant 13; abandoned

Several participants also reported having given up their hobbies. This was because of their reduced mobility and physical decline. Although some participants had just recently bought a vehicle, had a summerhouse or were still doing sports, this was no longer feasible. Because of this, they feel that they have lost a part of themselves.

### Body

In regard to the category of the body, reduced mobility, reduced appetite and insomnia were the most-cited factors

*“It could have been a little better. It is the appetite; I am not saying that the food is bad, and today I have already eaten a bit more. I also do not sleep well either. I'm overtired and altogether yes... that creates problems.”*

Participant 3; reduced appetite and insomnia

### Daily life functioning

The dependence on other people and the difficulty related to accepting help were two conflicting subjects. The participants reported getting older and therefore being more dependent on others. They needed, for example, more help from others to be able to live at home. The participants were aware of this need, and many found it difficult to accept such help, especially from strangers. This could be a reason why they remain unaided.

*“Yes, for the moment nothing is going to change, as long as she is well enough. When we notice that she is not doing well anymore, then we will call in more help. She wants to stay at home for as long as possible, and we want to respect that; but in that case, she has to accept more help.”*

Family member of participant 10; hard to accept help

### Quality of life

In this last category, the restriction of freedom was a prominent element. Many participants indicated that, due to these frequent hospital admissions, they had to give up many activities and were thus confronted with shortcomings in their daily life. This gave them the feeling that they had lost power over their own lives and that they cannot organize their own life according to their own wishes. The participants were also restricted in their freedom of movement due to their reduced mobility. These elements resulted in a reduced quality of life.

Participant*: “It is a fact that this reduced mobility is the result of physical complaints, especially with my back problems... I would like to work out, for example, but that is not possible because of physical problems. So yes, you always have to... Your environment is getting smaller and smaller.”*

Family of participant*: “A year ago, for example, she was still driving a car. That is no longer an option. She did her own shopping, but this is not possible anymore. She cannot walk far now. She has become less mobile.”*

Participant 11; freedom restriction

## Discussion

Our research revealed several triggers, underlying causes and contributing factors of frequent hospital admissions, as perceived by older people. Many of those perceived causes and factors - such as falling, medication errors, patient behavior (ignoring complaints, noncompliance, postponing consultation), lack of home care and family support, lack of medical follow-up and initiative, too-early discharge – are potentially avoidable or modifiable.

Some participants themselves indicated that solving these causes would lead to fewer hospitalizations. Moreover, several issues that are perceived as medical issues by the patients (e.g., lack of availability of their general practitioner) may in fact be related to issues with the system. Indeed, patients’ perceived causes of frequent hospital admission refer to well-known problems in the Belgian health care system, such as the overall dominant fee-for-service payment of general practitioners (as well as home nurses) and the lack of integration and teamwork. The almost exclusive fee-for-service model stimulates GPs and home nurses to maximally increase their individual contact with individual patients and thus discourages teamwork and care coordination for the patients [29]. To date, multidisciplinary teamwork with coordinated task delegation is almost absent in the daily practice of primary care. Innovative initiatives to stimulate care coordination, case management and neighborhood health care have recently been launched, but it is still too early to measure the impact of these programs [30, 31]. In well-functioning teams established for people with frequent hospital admissions, the home nurse or social worker could be asked to monitor issues that often lead to hospitalization. Thus, early intervention may solve the problem before hospitalization becomes inevitable.

Of note, frequent unplanned hospital admissions occur despite systematic comprehensive assessment and discharge planning at the geriatric hospital wards. Again, this observation may be explained by the isolated and uncoordinated care, this time between hospitals and primary care. At the time of discharge, hospitals in Belgium have little or no ability to affect the patients’ situation at home (and vice versa). However, in the last decade, different models and approaches to integrated care have been widely implemented and demonstrated in different settings. To date, there are no specific models for frequent flyers, but case management has been proposed as the most promising intervention [32]. This model would in fact reduce the number of hospital admissions and improve patient satisfaction [33]. Case managers play an essential role in the planning, processing and monitoring of the patient’s care, which involves the necessary health care services. Since communication and coordination are particularly important [34], transmural case management reduces the gap between primary and hospital care. In addition, there is less miscommunication between patients and care providers since the case manager acts as an interpreter. Previous research has indicated that case management increases the feeling of safety and well-being in patients and causes a decrease in psychological distress because it can compensate for issues such as missing social contacts and loneliness [32, 33].

**Fig. 3:**
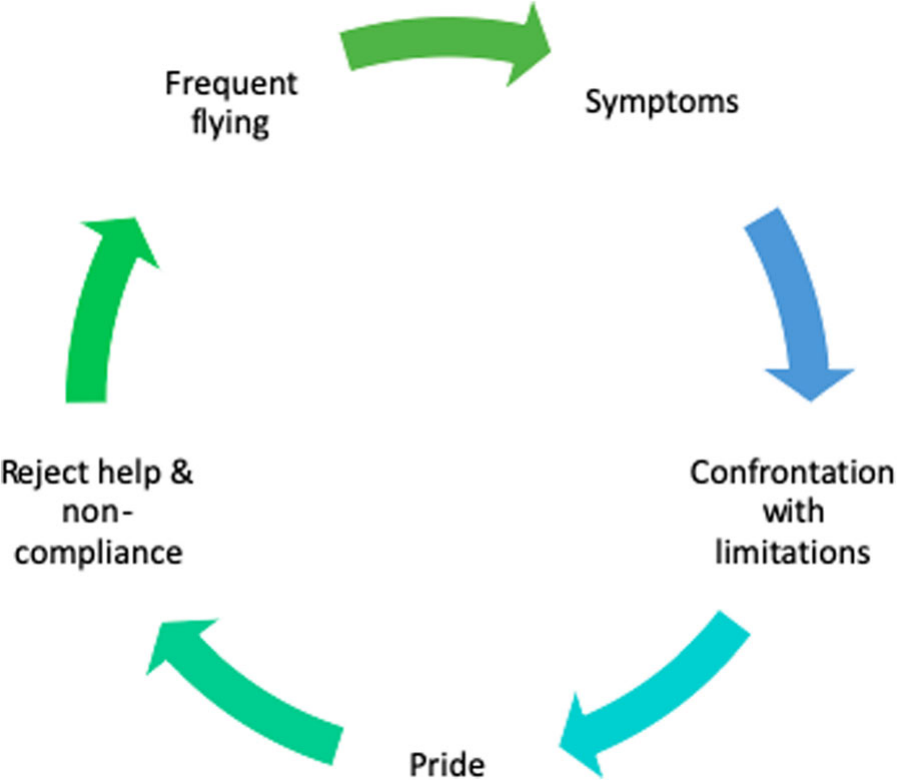
Vicious cycle due to the patient’s behavior

In accordance with the literature, all the participants in the study were people presenting with chronic multimorbidity and complex health needs [8, 35, 36]. Our findings suggest within this population a close interaction between perceived causes, contributing factors and consequences. These factors all affect each other, and it is difficult to view them separately. Most interestingly, the patients’ behavior and the ways in which they deal with their illnesses play a major role. Problems arise when patients start to ignore their symptoms. This may be due to the bad experiences of other patients, negligence on the part of the patient or the lack of medical insight. With the thought of “it will go away”, their conscience is appeased; however, the problem and a consultation with a physician are only postponed. Another reason why patients postpone their consultations is because of their pride. It is difficult to face the issue of physical and mental decline, and therefore, it is hard to accept help from others. Some patients want to be independent and want to prove their independence. The more they reject help, the more difficult it is to control the disease(s) and to remain compliant with the advised therapy. In this way, patients can remain stuck in a vicious circle (see Fig. 3).

This problem can be addressed by paying special attention to the patients’ ideas, concerns and feelings. In this case, it is important to find the appropriate wording for informing and motivating them. It is noticeable that older persons are frequently confronted with negative experiences, e.g., the death of a loved one, a poor health status, a family quarrel, etc. These situations lead to questions about the purpose of life, and within this consideration, the thought of death often arises. It may sound counterintuitive, but older persons may be more afraid of death than younger adults [37]. Their health status may explain this discrepancy. In our findings, loneliness, psychological distress, lack of sense making and having purpose in life are often mentioned as being more depressing than physical illness. Having meaning in life has been linked to better physical health and well-being, reduced mortality and greater happiness in old age [38]. The people who are most at risk of losing their purpose in life often have poor health, live alone, have a low socioeconomic status, are socially isolated, do not engage in any activities or are nonreligious [39–41]..

A regular comprehensive assessment of the patient’s health status and care needs, based on a validated and uniform approach like the interRAI tool [42] may allow for key decision making in the care of older persons [43, 44]. Our results suggest that it is also fundamental that caregivers develop the competence to recognize the spiritual and/or existential dimension in patients in their daily work environment. The concept of positive health can be used to support the caregiver-patient relationship and the shift from institutional care to adaptability and self-management in elderly individuals [27, 28]. Dealing with the spiritual and/or existential dimensions of patients’ lives can be done during the process of Advanced Care Planning (ACP). ACP has received increased attention in recent years. It can be defined as *“A process that supports adults at any age or stage of health in understanding and sharing their personal values, life goals, and preferences regarding future medical care (…)”* [45]. Several studies, mostly conducted in the palliative care setting, have shown that advanced care planning is effective in reducing frequent hospital admissions, emergency department visits and 30-day readmissions of frail and older people [46–49]. However, reducing hospital admissions and costs may never be the sole aim of ACP.

Few articles have been published that have explored the causes of frequent hospital admissions from the patient’s point of view. When looking at the available findings, there are some similarities. For example, in the study of O’Leary et al., the lack of family support, financial barriers, pain and limitations in activities are observed [21]. In addition, the participants in the study of Liu et al felt safe in a hospital. These outcomes correspond to our data on being pampered during admission and trusting the hospital [22]. Those articles, however, have not made a subdivision between causes, contributing factors and consequences.

While some of our findings reinforce those of previous studies, others are contradictory. In the study of O’Leary et al, the participants had no desire to go to the hospital and did not describe being in the hospital as a favorable or positive experience [21]. However, this is not the case in our study. One participant admitted having more confidence in specialists/hospitals than in general primary care. Moreover, many participants reported that their hospital stay was a reassuring and appeasing experience. Furthermore, the study of Liu et al concluded that outside-hospital support would not decrease hospital admissions, while in our study, the lack of support (cause) and inadequate transmural care (contributing factor) emerged as strong factors [22]. Hence, according to our results, improved support, i.e., better teamwork between health care providers and community services outside the hospital, may reduce hospital dependency.

### Limitations

This study also had some limitations. First, our participants were recruited in a single large academic hospital, while the patient population differs across hospitals. Additionally, some patients may have been missed, namely, those who were hospitalized 4 times or more, but not always at the same UZ Leuven hospital. Furthermore, the participants were only recruited from the geriatric department. These factors limit the study’s generalizability to patients admitted to community hospitals and other departments. The participants were also interviewed during their hospitalization. This situation expresses a situation of great vulnerability, which may have had an impact on the participants’ answers.

Second, patients with poor hearing function, those with a known diagnosis of dementia, those with delirium, those who were non-Dutch speaking, those who were DNR3 and those having no speech function were excluded from the study. However, it is possible that patients with such characteristics have different backgrounds and perspectives that could have enriched the data.

Finally, interviews with family members were incorporated, which provided additional insights and clarifications into the reported health care experiences. This only happened when a family member was present at the time of the interview; however, for future studies, it is recommended to also examine the perspectives of family members.

## Conclusion

Frequent hospital admissions may result from complex interactions between patients’ physical and mental condition, attitude, values and social situation, together with issues related to the provision of care. Therefore, frequent hospital admissions may be considered an indicator, i.e., a ‘red flag’, of patients’ physical, mental, spiritual and social deprivation in their home situation. Those patients should be ‘flagged’ because they are in need of special attention from a transmural, multidisciplinary team of social and health care workers. This team should provide a holistic and patient-centered approach, including case management and a program of advanced care planning. This approach may also reduce the number of hospital admissions, which is a hypothesis that is still in need of further research.

## Supplementary information

**Additional file 1: Supplementary File 1.** The filled in checklist of the ‘Consolidated Criteria for Reporting Qualitative Research’.

**Additional file 2: **S**upplementary File 2.** Research team and reflexivity.

**Additional file 3: Supplementary File 3.** Interview guide (translated ino English from the Dutch language).

**Additional file 4: Supplementary File 4.** Overview of contributing factors.

## Data Availability

All the interviews were transcribed into text files. These text files containing the transcripts have been delivered to the promoter (corresponding author), who has placed them in a secured server. Anonymized transcriptions are available at request.

## Abbrevations

ACP: Advanced Care Planning
COREQ: Consolidated Criteria for Reporting Qualitative Research
DNR: Do-Not-Resuscitate
ED: Emergency Department
ENT: Ear, Nose, Throat
EU: European Union
QUAGOL: Qualitative Analysis Guide of Leuven
UZ: Universitair Ziekenhuis

## References

[1] European Commission, OECD. Health at a Glance: Europe 2018 State of health in the eu cycle 2018. doi:10.1787/health_glance_eur-2018-en..

[2] Friebel R. Trends in the number of English NHS hospital admissions, 2006 to 2016 | The Health Foundation. https://www.health.org.uk/chart/chart-trends-in-the-number-of-english-nhs-hospital-admissions-2006-to-2016. (Accessed 25 Jun 2020).

[3] Braet A, Weltens C, Sermeus W (2015). Risk factors for unplanned hospital re-admissions: a secondary data analysis of hospital discharge summaries. J Eval Clin Pract.

[4] Mohammed MA, Mant J, Bentham L (2006). Process of care and mortality of stroke patients with and without a do not resuscitate order in the west midlands, UK. Int J Qual Health Care.

[5] Capp R, Kelley L, Ellis P, et al. Reasons for frequent emergency department use by Medicaid enrollees: a qualitative study. Acad Emerg Med. 2016. 10.1111/acem.12952. 10.1111/acem.12952 26932230

[6] Althaus F, Paroz S, Hugli O (2011). Effectiveness of interventions targeting frequent users of emergency departments: a systematic review. AnnEmergMed.

[7] Blumenthal D, Chernof B, Fulmer T (2016). Caring for high-need, high-cost patients - an urgent priority. N Engl J Med.

[8] Szekendi MK, Williams MV, Carrier D (2015). The characteristics of patients frequently admitted to academic medical centers in the United States. J Hosp Med.

[9] McCarthy CP, Pandey A (2018). Predicting and preventing hospital readmissions in value-based programs. Circ Cardiovasc Qual Outcomes.

[10] Hospital Readmission Reduction | CMS. https://www.cms.gov/Medicare/Quality-Initiatives-Patient-Assessment-Instruments/Value-Based-Programs/HRRP/Hospital-Readmission-Reduction-Program. (Accessed 1 Jun 2020).

[11] Zuckerman RB, Sheingold SH, Orav EJ (2016). Readmissions, observation, and the hospital readmissions reduction program. N Engl J Med.

[12] Tricco AC, Antony J, Ivers NM (2014). Effectiveness of quality improvement strategies for coordination of care to reduce use of health care services: a systematic review and meta-analysis. CMAJ.

[13] McCants KM, Reid KB, Williams I (2019). The impact of case management on reducing readmission for patients diagnosed with heart failure and diabetes. Prof Case Manag.

[14] Finlayson K, Chang AM, Courtney MD (2018). Transitional care interventions reduce unplanned hospital readmissions in high-risk older adults. BMC Health Serv Res.

[15] Braet A, Weltens C, Sermeus W (2016). Effectiveness of discharge interventions from hospital to home on hospital readmissions: a systematic review. JBI Database Syst Rev Implement Rep.

[16] Luben R, Hayat S, Wareham N (2020). Usual physical activity and subsequent hospital usage over 20 years in a general population: the EPIC-Norfolk cohort. BMC Geriatr.

[17] Iglesias K, Baggio S, Moschetti K (2018). Using case management in a universal health coverage system to improve quality of life of frequent emergency department users: a randomized controlled trial. Qual Life Res.

[18] Fabbri E, Zoli M, Gonzalez-Freire M (2015). Aging and multimorbidity: new tasks, priorities, and Frontiers for integrated Gerontological and clinical research. J Am Med Dir Assoc.

[19] Abdi S, Spann A, Borilovic J (2019). Understanding the care and support needs of older people: a scoping review and categorisation using the WHO international classification of functioning, disability and health framework (ICF). BMC Geriatr.

[20] Herr M, Arvieu JJ, Aegerter P (2013). Unmet health care needs of older people: prevalence and predictors in a French cross-sectional survey. Eur J Pub Health.

[21] O’Leary KJ, Chapman MM, Foster S (2019). Frequently hospitalized patients’ perceptions of factors contributing to high hospital use. J Hosp Med.

[22] Liu T, Kiwak E, Tinetti ME (2017). Perceptions of hospital-dependent patients on their needs for hospitalization. J Hosp Med.

[23] Fisher KA, Smith KM, Gallagher TH (2017). We want to know: Eliciting hospitalized patients’ perspectives on breakdowns in care. J Hosp Med.

[24] Tong A, Sainsbury P, Craig J (2007). Consolidated criteria for reporting qualitative research (COREQ): a 32-item checklist for interviews and focus groups. Int J Qual Heal Care.

[25] Longman JM, I Rolfe M, Passey MD, et al. (2012). Frequent hospital admission of older people with chronic disease: a cross-sectional survey with telephone follow-up and data linkage. BMC Health Serv Res.

[26] Dierckx de Casterle B, Gastmans C, Bryon E (2012). QUAGOL: a guide for qualitative data analysis. Int J Nurs Stud.

[27] Huber M, André Knottnerus J, Green L, et al. How should we define health? BMJ. 2011;343. 10.1136/bmj.d4163. 10.1136/bmj.d4163 21791490

[28] Huber M, Van Vliet M, Giezenberg M, et al. Towards a ‘patient-centred’ operationalisation of the new dynamic concept of health: a mixed methods study. BMJ Open. 2016;6. 10.1136/bmjopen-2015-010091. 10.1136/bmjopen-2015-010091 PMC4716212 26758267

[29] Russell GM, Miller WL, Gunn JM (2018). Contextual levers for team-based primary care: lessons from reform interventions in five jurisdictions in three countries. Fam Pract.

[30] Interprofessionele samenwerking: enkele termen uitgelegd - Eerstelijnszone. https://www.eerstelijnszone.be/interprofessionele-samenwerking-enkele-termen-uitgelegd. (Accessed 13 Jun 2020).

[31] Home | Zorgzaamleuven. https://www.zorgzaamleuven.be/. (Accessed 13 Jun 2020).

[32] Hudon C, Chouinard MC, Dubois MF, et al. Case management in primary care for frequent users of health care services: a mixed methods study. Ann Fam Med. 2018. 10.1370/afm.2233. 10.1370/afm.2233 PMC5951252 29760027

[33] World Health Organization Regional Office for Europe W. Integrated care models: an overview Working document. 2016. https://www.euro.who.int/__data/assets/pdf_file/0005/322475/Integrated-care-models-overview.pdf.

[34] Julian K. Case Management Definition. https://www.investopedia.com/terms/c/case-management.asp. (Accessed 25 Jun 2020).

[35] Althaus F, Stucki S, Guyot S (2013). Characteristics of highly frequent users of a Swiss academic emergency department: a retrospective consecutive case series. Eur J Emerg Med.

[36] Legramante JM, Morciano L, Lucaroni F (2016). Frequent use of emergency departments by the elderly population when continuing care is not well established. PLoS One.

[37] Cicirelli VG (2002). Fear of death in older adults: predictions from terror management theory. Journals Gerontol Ser B Psychol Sci Soc Sci.

[38] Boyle PA, Barnes LL, Buchman AS (2009). Purpose in life is associated with mortality among community-dwelling older persons. Psychosom Med.

[39] Pinquart M (2002). Creating and maintaining purpose in life in old age: a meta-analysis. Ageing Int.

[40] Clarke P, Marshall V, Black SE (2002). Well-being after stroke in Canadian seniors: findings from the Canadian study of health and aging. Stroke.

[41] Krause N, Hayward RD (2012). Religion, meaning in life, and change in physical functioning during late adulthood. J Adult Dev.

[42] interRAI. https://www.interrai.org/. (Accessed 13 Jun 2020).

[43] Schluter PJ, Ward C, Arnold EP (2017). Urinary incontinence, but not fecal incontinence, is a risk factor for admission to aged residential care of older persons in New Zealand. Neurourol Urodyn.

[44] Sciannameo V, Berchialla P, Orsi E (2020). Enrolment criteria for diabetes cardiovascular outcome trials do not inform on generalizability to clinical practice: the case of glucagon-like peptide-1 receptor agonists.

[45] Sudore RL, Lum HD, You JJ (2017). Defining Advance Care Planning for Adults: A Consensus Definition From a Multidisciplinary Delphi Panel. J Pain Symptom Manage.

[46] Brumley R, Enguidanos S, Jamison P (2007). Increased satisfaction with care and lower costs: results of a randomized trial of in-home palliative care. J Am Geriatr Soc.

[47] Takahashi PY, Haas LR, Quigg SM (2013). 30-day hospital readmission of older adults using care transitions after hospitalization: a pilot prospective cohort study. Clin Interv Aging.

[48] Chen YR, Yang Y, Wang SC, Chou WY, Chiu PF, Lin CY, et al. Multidisciplinary care improves clinical outcome and reduces medical costs for pre-end-stage renal disease in Taiwan. Nephrology. 2014;19:699–707. 10.1111/nep.12316. PMC4265277 25066407

[49] Chen CY, Thorsteinsdottir B, Cha SS (2015). Health care outcomes and advance care planning in older adults who receive home-based palliative care: a pilot cohort study. J Palliat Med.

