# Supplementary material for: Patients’ perceptions of frequent hospital admissions: a qualitative interview study with older people above 65 years of age
Source: BMC Geriatr. 2020 Sep 7;20:332. doi: 10.1186/s12877-020-01748-9 (PMC7487888; doi:10.1186/s12877-020-01748-9)
Supplement: Supplementary file 2 — Additional file 2: Supplementary File 2. Research team and reflexivity. [file 12877_2020_1748_MOESM2_ESM.docx]

## Supplementary File 3: Research team and reflexivity

| **Personal Characteristics** | | |
| --- | --- | --- |
|  | Cindy Miaolin Huang (CMH) | Carolien Van der Borght (CVDB) |
| Interviewer | Conducted 6 interviews  Coded 7 interviews | Conducted 7 interviews  Coded 6 interviews |
| Credentials | MD | |
| Occupation | Pharmacist in hospital  Student | Audiologist  Student |
| Sex | Female | |
| Training | Read the book “Handboek kwalitatieve onderzoeksmethoden” by Dimitri Mortelmans.  Followed an online course “Introduction to research methods in health” by Marieke Kroezen and Luk Bruyneel | |
| **Relationship with participants** | | |
| Relationship established | No relationship required to commence study | |
| Participant knowledge of the interviewer | Their occupation and master’ education | |

## 
