# Supplementary material for: Patients’ perceptions of frequent hospital admissions: a qualitative interview study with older people above 65 years of age
Source: BMC Geriatr. 2020 Sep 7;20:332. doi: 10.1186/s12877-020-01748-9 (PMC7487888; doi:10.1186/s12877-020-01748-9)
Supplement: Supplementary file 3 — Additional file 3: Supplementary File 3. Interview guide (translated ino English from the Dutch language). [file 12877_2020_1748_MOESM3_ESM.docx]

# Supplementary File 2: Interview guide - 'frequent flyers, translated from Dutch

Name interviewer:

Patient number:

Date of interview:

Duration of interview:

## Introduction of the study (reading together the information letter)

Instructions for the investigators

1. Introduce yourself: “Who are we?”

2. Explain what the purpose of this investigation is.

3. Ensure anonymity and confidentiality

4. Request permission for recording

5. Ask to sign informed consent or to check that it has been signed.

6. Ask if the patient has any questions before we start the interview.

7. Do not use abbreviations or jargon.

8. Estimate for each question as well:

a. Whether the question really should be asked

b. Or that you can implicitly deduce from the situation that it is a problem so that the question is adjusted e.g. I see that you are in a wheelchair, do you manage to move anywhere in the house?

c. Whether it is not threatening

d. Whether the answer to the question can be deduced from previous answers

## T1: Introductory questions

Q1: How are you doing?

Q2: What were you/are you admitted for?

## T2: Causes/factors of repeated hospitalization

Q3: Why are you hospitalized several times?

Q4: Did ... play a role in the fact that you have been hospitalized several times?

○ Medical care (e.g. general practitioner, specialist, GP, pharmacist, informal caregivers, children, etc.)

○ The patient himself

○ Family and friends

○ Your current health situation

○ Financial situation

○ Medication

Q5: Were there less good experiences in general?

○ How could it go better?

Q6: How do you think hospital admissions could be avoided in the future?

○ Who plays a role in this?

○ What would you like to change?

## T3: How do they experience the causes of repeated hospitalizations and their impact on their current condition (physical, psychological-emotional and social?)?

Q7: Have these causes had an influence on your...

○ Physical condition? E.g., Can you no longer carry out certain activities that were previously possible?

○ Mental/moral status? What about your morals?

○ Social life? What about social life?

Q8: How does your family feel about it?

Q9: What was the impact on your autonomy?

Q10: Was it difficult to resume your daily routine after admission?

## T4: Experience of care before, during and after repeated hospitalizations

### Questions on "experience before repeated hospitalization"

Q11: What kind of care did you receive before the hospital admissions (pharmacist, GP, home nurse, family, physiotherapy, home care,...)?

Q12: What did you think of the care you received before the hospital admissions?

○ Did you receive sufficient information (e.g. disorder, medication, treatment,...)?

○ What went well ? What went less well?

Q13: How did you get to the hospital? (Referral to a GP, consultation, emergency, own initiative,...)

○ If you need urgent care or something is not right, is it easier to see a GP or do you opt directly for a hospital visit?

○ Is the GP easily accessible or what could be better?

○ How can the care in your area be optimized?

Q14: What do you think of the transition from home to hospital?

○ What went well and what went less well?

### Questions about "experience during repeated hospital admissions

Q15: What kind of care did you receive during admission?

Q16: What did you think of the care you received during hospitalization?

○ Did you receive sufficient information?

○ What went well and what went wrong?

### Questions about "experience after the repeated hospitalizations

Q17: What kind of care did you receive after the admission?

Q18: What did you think of the care you received after hospitalization?

○ Did you receive sufficient information?

○ What went well and what went wrong?

Q19: How did you get from hospital to home?

Q20: What do you think of the transition from discharge to home?

○ What went well and what went less well?

○ What information did you get from the hospital? Was it sufficient and clear?

○ Was everything clear what you had to do at home?

○ Who will be there for follow-up?

Q21: Ask if the patient has any questions or would like to add anything else to the interview
