# Supplementary material for: Patients’ perceptions of frequent hospital admissions: a qualitative interview study with older people above 65 years of age
Source: BMC Geriatr. 2020 Sep 7;20:332. doi: 10.1186/s12877-020-01748-9 (PMC7487888; doi:10.1186/s12877-020-01748-9)
Supplement: Supplementary file 4 — Additional file 4: Supplementary File 4. Overview of contributing factors. [file 12877_2020_1748_MOESM4_ESM.docx]

## Overview of contributing factors

| **Medical** | | | **Non-medical** | **Patient** |
| --- | --- | --- | --- | --- |
| General practitioner | | | Family | Behaviour |
| Insufficient care    -Lack of follow-up  -Lack of information  -Lack of initiative  -Lack of knowledge  -Wrong estimation  -Professional jargon  -Lack of patient involvement | Availability    -Too busy  -Search for suitable GP | Miscommunication | -Lack of help  -Lack of contact  -Family quarrel  -Disagreement about care  -Loss of loved ones  -Widowhood | -Fear of treatment because of bad experiences from others  -Negligent  -Fear of asking clarification  -No care seeking without consent GP  -Proud  -Xenophobia  -Lying to the physician  -Easily satisfied |
| Nursing home | | | Home care | Physical |
| Insufficient care    -Lack of initiative  -Wrong estimation  -Medication errors  -Lack of patient involvement | Bad coordination | Miscommunication | -Lack of home care | -Forgetfulness  -Underweight  -Deteriorating complaints  -Reduced mobility  -Reduced appetite  -Physically weak |
| Hospital | | | Social environment | Finances |
| Insufficient care  -Lack of communication  -Lack of information  -Not treating the problem  -Lack of patient involvement | Fragmented care    -Too many treating doctors  -Shifted responsibility  -Overreporting  -Double therapy  -Over-specialisation  -Lack of communication between specialists | Others    -Pampering/ pleasant  -Too crowded  -Trust | -No friends  -Lack of neighbourly contact  -Lack of other social contacts | -Health care costs  -Health insurance |
| Specialist | | |  | Lifestyle |
| Insufficient care    -Lack of information | Availability    -Difficult to make an appointment  -Too busy  -Long waiting time | Miscommunication |  | -Alcohol  -Smoking  -Too much free-time |
| Drugs | | |  | Others |
| -Side effects  -Polypharmacy  -Lack of drug knowledge | | |  | -Cause unknown  -Lack of medical knowledge  -Misconceptions |
| Transmural care | | |  |  |
| -No proper care after dismissal  -Insufficient information on dismissal letter | | |  |  |
